# Electrical safety in a hospital setting: A narrative review

**DOI:** 10.1016/j.amsu.2022.103781

**Published:** 2022-05-17

**Authors:** Loganathan Salvaraji, Mohammad Saffree Jeffree, Khamisah Awang Lukman, Sahipudin Saupin, Richard Avoi

**Affiliations:** a Public Health Medicine Department, Faculty of Medicine and Health Science, Universiti Malaysia Sabah, Kota Kinabalu, Sabah, Malaysia; b Centre for Occupational Safety and Health, Universiti Malaysia Sabah, Kota Kinabalu, Sabah, Malaysia

## Abstract

Electrical hazard is a significant threat in hospitals that require sustainable assessment and improvement. Workplace assessment and medical surveillance systems are often overlooked, and employees fail to comply with the necessary measures that have been instructed at the workplace. The incidents are attributed to a lack of knowledge regarding occupational safety and health and a lack of awareness regarding electrical hazards importance. The consequences of neglecting these assessments may result in health deterioration and low work quality and may also cause disability and impairment. This review describes occupational safety and health perspective about electrical hazards in hospital settings. Further preventive measures were recommended to outline practical and feasible steps resulting in a safer and healthier working environment.

## Highlights

• Electric hazard is a significant threat in hospitals that require sustainable assessment and improvement.
• This is attributed to a lack of knowledge regarding occupational safety and health and a lack of awareness regarding its importance.
• The consequences of neglecting these assessments may result in health deterioration, low work quality, and may also cause impairment.
• Hospitals should adopt workplace assessments to improve the overall health of all the workers, and quality in health service.

## 1 Introduction

In occupational health and safety, a hazard is commonly defined as any potential source that can damage, harm, or have adverse health effects on a worker [1]. It is also a condition where workers can contact energized equipment or a conductor [2]. Hospital is equipped with a plethora of electrical apparatus. These devices vary according to location and function. In an office or administrative counter, the staff uses a computer, printer, and photostat machine to register patients, meet preparation, and human resource management. On the other hand, medical devices such as infusion pumps, suction machines, and physiotherapy equipment are used for medical treatment [3]. During the operational procedure, a patient will be under a ventilation machine. Medical instruments such as operation endoscopies, laser devices, and radiology scanning machines were applied as part of interventional treatment [4].

Large electrical supplies are found in the radiology department, such as X-ray machines, computerized tomography, and magnetic resonance imaging [5]. For diagnostic purposes, sampling is run under an electronic machine to get particular biological markers such as full blood count, urea and creatinine, antibodies, and microorganisms culture [6]. Working near or with an electrical device is dangerous and can be fatal. Contact with electrical current may cause negative effects on the human body. The electric circuit can be underrun in the human body, leading to organ injury and failure. Blast from electrical equipment can cause burn, perforated tympanic membrane, and multiple trauma [7]. Contact between human and current or blast happens when there is a defect in the device such as frayed electrical cord, damaged plug, damaged wires, and missing prong [1]. Besides that, unsafe acts by the workers also increase the risk for an electrical accident, such as holding wire, multiple extension plugs, and uncovering electrical wire. Surgical smoke created during diathermy also can trigger respiratory symptoms, carcinogenicity, mutagenicity, and infection [8].

A fire incident in Johor Bahru, Malaysia at a government hospital killed six patients in the intensive care unit (ICU) [9]. The fire and rescue department declared that the fire was due to faulty wiring. ICU is affected because of oxygen gas continuously supplied to the patient. The electric sparks reacted with oxygen and blazed fast throughout the unit. The curtains and beds burned and produced smoke that delayed the fire and rescue activity further. Staff suffered mild suffocation due to smoke inhalation. Firefighters took almost 2 h to put down the fire and manage to control the situation. Patients from the building were evacuated to nearby hospitals and private hospitals. Another fire incident in Kuala Lumpur, Malaysia reported due to a short circuit at a forensic department store. The electric spark caused plastic and papers to catch on fire in the store. The fire and rescue team vigilantly arrived and saved two workers trapped in the storeroom. Meanwhile, patients from the nearby ward were transferred to other buildings. However, there were no death or injury was reported. The hospital team managed to transport oxygen tanks before the fire expanded to another storeroom. The rescue team helped shift the dead bodies because the power supply was cut off [10].

There was no healthcare worker reported died because of electric shock or blast. Nevertheless, the safety of healthcare workers cannot be taken for granted, and there is always the risk for such incidents to happen. An electrical safety program is essential to implement and maintain routine core business at the hospital. The program's objective is to prevent near-missed incidents that lead to loss of productivity and costs. There are several benefits to the organization by practicing electrical safety and prevention efforts. The electrical prevention measures ensure the safety of the workers is guaranteed or the risk of an electrical accident is minimized.

Injury from an electrical accident can lead to severe complications and death. Some may even end up with a disability requiring long-term medical support. Properly maintaining these instruments from time to time will ensure good condition and functioning for long time use. Implementing prevention measures able reduce the risk of indirect detrimental effects to the rescue team. The organization's reputation is preserved by executing prevention electrical measures. A worker's death or injury represents the seriousness and commitment of the organization ensuring a safe workplace. Minimal accidents at the workplace reflect that the safety and health committee and workers adhere to the safety procedures. It is important to establish a necessary measure to lessen the risk of the unfortunate incident occurring. Employers and employees must work hand in hand by sharing opinions and expertise to form a best practice outline at the site.

## 2 Mechanism of electrical induced injuries

Electric current can cause a significant defect to the human body when in contact. The post effect relies on the magnitude of energy, resistance, type of current (Alternating Current or Direct Current), current pathway, and length of exposure [11]. A higher magnitude of the wind in contact with the human body will cause severe systemic effects and tissue damage. Generally, high voltage is more the 1000 V and low voltage less than 1000 V. Physiologically, it is known that Alternating Current (AC) is far more dangerous than Direct Current (DC) [12]. AC will trigger tetanic muscle contraction, and the victim cannot execute from the electric source. Hence, this increases the duration and intensity of the electric current. The victim will experience respiratory arrest due to diaphragm and intercostal muscles tetany. Subsequently, frequent current delivery will extend to the myocardium and interrupt the cardiac cycle, precipitating ventricular fibrillation, a life-threatening condition. On the other hand, Direct Current (DC) involves a single violent muscle contraction and thrusting the victim away from the source. The complication depends on the voltage that has been exposed. Higher voltage is associated with more significant mortality and morbidity. Besides that, the current pathway also plays a role in the organ involved. If the current passes through the head or thoracic organ, the condition may be fatal. Brain injury or seizure can develop respiratory arrest and paralysis. Meanwhile, the transthoracic currents stimulate cardiac arrhythmia, direct cardiac damage, and respiratory arrest. Local damage of the tissues such as muscle, tendon, and skin will develop tissue oedema. Further, this condition will compress the vascular and nervous supply to the organ, known as compartment syndrome. For example, during a surgical procedure, surgeons use diathermy for many purposes, such as controlling bleeding and cutting tissues. The circuit used primary voltage and referenced to earth by electrodes. If the pad is loose or wrongly placed, the current will find an alternative path to the planet causing electrical injury such as burns [13]. Subjects expert matter have agreed that there are three mechanisms in electric-induced injury is [14] direct tissue damage (cell membrane alteration resting potential and muscle tetany), electrical energy conversion to thermal energy leading to tissues destruction and necrosis, blunt trauma, or blow causing fall and violent muscle contraction, and smoke from the electrical device used in surgery such as diathermy can cause respiratory irritation or skin allergy.

## 3 Electrical hazards in hospital setting

A systematic review study on occupational hazards among hospital resuscitation team highlighted typical electrical injury occurred during defibrillation to revert cardiac rhythm in ventricular fibrillation and pulseless ventricular tachycardia rhythm. There was 29 health effect reported due to defibrillator use. Upon investigation, three incidents were reported due to equipment defects (e.g., paddles crack, wrong discharge)—four of the incidents were improper training and maintenance of the device (e.g., accidental discharge). The unexpected shock was reported in fifteen incidents. Other incidents are due to contact with a patient or stretcher during defibrillation. HCWs complain of burning and tingling sensation. A case report of electrical injury during defibrillation was shared, mentioning the shock was delivered during the cardiac massage. The massager sustained electric shock and was unable to recover for 30 min. In another incident, the massager was thrown away during defibrillation resulting in neck and back pain [15].

A retrospective study at teaching hospital was conducted in an endoscopic room to study exposure above the floor wires. This study reviews 110,000 endoscopic procedures and orthopaedic injuries for the past five years. It was highlighted that three endoscopic personnel experienced severe musculoskeletal injuries, including metacarpal fracture, rib fracture, and ankle sprain. Hence, this condition led to lost days of work (mean 9.3 ± 11.0 (SD) days) and light-duty (mean 41.7 ± 31.8 days). The hospital revealed a mean of 35.3 ± 7.5 cards, wires, or tubing per endoscopy procedure room; almost all wire was exposed to personnel before remediation [16]. However, electrical shock or electrocution among personnel in the endoscopy room was not mentioned.

A study in United Kingdom about thermal injury among patients during Magnetic Resonance Imaging (MRI) was analysed by Dempsey et al. [17]. This study involved secondary data analyses from injury or accidents reported to the Centre of Devices and Radiological Health, United State Food and Drug Administrations. The study reviews the burn association with electrocardiography (ECG) monitoring, pulse oximetry, and coils. Eighty-six case was reported with ECG monitoring. Most burn areas are underneath the electrode. The patient experienced third-degree burn (23%), second-degree burn (12%), and others were not specified (65%). The cause of the burn is due to the cable loop, the proximity of cable to MRI, electrode characteristics, obese patients, and higher power investigations.

On the other hand, thirty-six cases were reported associated with pulse oximetry. The common burn site is at the fingers. Thirteen percent (13%) of cases experience a third-degree burn, and fifty-six percent (56%) had second-degree burns. Only four patients sustained a burn to the toe. In MRI, a coil is used to hold the patient while conducting the procedure. There are 23 cases of electrical injury and accidents reported due to the ring. The coiled used are Cervical Thoracic Lumbar (CTL), Shoulder, Cervical Spine (CS), and Knee. CTL coil causing a burn to thumb & thigh (1 case), hand & buttock (4 cases), and shoulder & arm (3 cases). Two cases sustained burn at arm due to shoulder coil. Four cases sustained burn at neck caused by Cervical Spine coil and two incidents of knee coil causing burn at the leg. Besides that, one patient sustained skin burn when in contact with a coiled cable. Six other burn cases have not specified the cause of the incident [17].

A 60% of 137 respondents experienced acute health effects due to surgical smoke inhalation in Hospital Serdang, Malaysia Predominate symptoms mentioned are respiratory irritation (40.0%), headache (27.4%), eye irritation (20.6%), and asthma-like symptoms (12.0%). Assisting in surgery was a significant predictor of at least one acute health effect inhalation of smoke (AOR = 2.7, 95% CI = 1.1, 7.0). Four factors were associated with at least one acute health effect: department, surgery role, employment duration, and the number of surgeries conducted or assisted per week [18].

Electrical safety awareness was tested among 401 healthcare workers in the Tanta university hospital, Egypt. Healthcare workers consisted of physicians, technicians, nurses, and others. Almost 80% of them admit that a licensed electrician installs the electrical equipment. Only 60% of them reported the electrical device was checked frequently. Half of the workers informed that wire, cables, plugs, and sockets were satisfactory. Two-thirds of workers (66%) were not given a briefing of electrical hazards in the hospital [19] (see Table 1).

**Table 1:** Health effects due to electrical hazard exposure in a healthcare facility.

Author	Title	Medical equipment/device	Health effect
Vindigni et al., 2017 [15]	Hospital resuscitation teams: a review of the risks to the healthcare worker	Defibrillator	- burn - tingling sensation - musculoskeletal pain
Cappell, 2010 [16]	Injury to Endoscopic Personnel from Tripping over Exposed Cords, Wires, and Tubing in the Endoscopy Suite: A Preventable Cause of Potentially Severe Workplace Injury	Endoscopy equipment's and wires	- bone fracture - joint sprain
Dempsey& Condon, 2001 [17]	Thermal Injuries Associated with MRI	Coil (MRI) ECG Pulse oximetry	- burn
Titi Rahmawati & Mohd Fikri, 2019 [18]	Health Effects of Surgical Smoke And Its Associated Factors Among Perioperative Healthcare Workers In Hospital Serdang	Electrosurgery device in operation theatre	- respiratory irritation - headache - eye irritation - asthma-like symptoms

## 4 Electrical safety and preventive measures in hospital

Risk control defines as action to eliminate or inactivate hazards. They were implementing measures as practicable during the work process to minimize the risk of adverse health effects or property and environmental damage. Traditionally, few types of control are presented in the hierarchy of control, namely elimination, substitution, isolation, engineering, administrative, and personal protective equipment (PPE). Hence, management's workplace assessment is evaluated according to the hierarchy [20]. The wiring is cord properly. Sufficient space of electrical equipment and worker able to move freely. Labelling of electrical equipment that can be visible. Ensure all electrical equipment is far from water sources. Remove unnecessary items or damaged equipment from the bay. Shift working can reduce exposure to hazards. Awareness and training for staff about electrical hazards, for example, advice staff not to touch electrical equipment with wet hands. Continue Medical Education (CME) on electrical hazards and real case scenarios for sharing experience. Establish occupational safety and health policy in the department and ensure workers adhere to items mentioned in the policy. Regular risk assessments to most units and check the condition of the electrical circuit and medical equipment such as cardia monitor, infusion pump, suction, ventilation, etc. Regular update and discussion of electrical hazard status during OSH committee meeting.

OSH prevention requires a holistic approach involving employer and employee. Both parties work hand in hand to avoid accidents, injuries, incidents minimize or eliminate safety and health risk. It is the responsibility of the employee to provide a safe and healthy working condition. Department of Labour United States recommends a few suggestions to implement effective control and prevention. Workers should be involved because they best understand the environment that creates hazards and control it. The hierarchy of management needs to be implemented and evaluated. Establish a hazard control plan to guide selection for emergency and non-routine activities to protect workers. Review new methods or technology that can protect workers, more reliable or less costly. For example, the endoscopic room has a compact space with a television monitor, scope, power supply, disinfectant, and patient bed. Besides that, this procedure requires a minimum of 4 healthcare personnel, including a surgeon, medical officer, house officer, medical assistant, and nurse. Therefore, OSH prevention programs among health personnel in the endoscopy room should include a database of healthcare workers, including their details, job descriptions, workplace accidents, or near missed. OSH policy of the hospital or department. Review OSH committee meeting and discussion. Medical surveillance for healthcare workers according to hazard exposure. Awareness of electrical hazards and fire.

Hospital is well known for biological hazards. Nevertheless, the recent incident of fire breakout in hospitals highlighted that electrical hazard is the top crucial issue in a hospital setting. This is because it threatens the lives of occupants and property damage that costs millions of dollars. Hospital management needs to identify a high-risk area that potentially can cause electrical injury or accident. This area can be analysed according to usage and purchasing of high voltage machines such as radiology, operation theatre, intensive care unit, endoscopy room, etc. Electrical hazards can cause devastating impacts in hospitals and identify as the main culprit to provoke fire. This has been revealed in most of the forensic reports by the fire and rescue team. They confessed damaged wire or short circuits had a significant cause of the fire. Hospitals highly the potential to have a fire because they are equipped with fire-prone material such as oxygen tanks, medication, reagents for laboratory tests, treatment with plastic, etc. Besides that, electrical hazards can seriously affect workers and patients in the ward. The severity of the condition depends on electrical factors such as voltage, current pathway, type of current, etc. Healthcare workers are at risk of sustaining at least second-degree burns when in contact with electrical current. When the current passes through the heart or brain, the risk increase can lead to cardiac arrhythmia, respiratory arrest, and brain death.

In most cases, the victim needs to undergo limb amputation and live with a disability. Hospital management must take the responsibility to ensure working conditions or environment must be safe for employers and clients. Proportionally, employers and clients must adhere to the hospital's OSH Policy. This includes following safety procedures and not attempting any high-risk attitude. OSH committee must play a role in assessing electrical hazards and be proactive to solve the problem as soon as possible. The recommendation for managing electrical risks is the space must be sufficient to place electrical devices and movement of occupants. This will reduce the chance of contact with electrical current. Healthcare workers can manipulate the device conveniently and focus more to prevent errors. Replace electrical device with device better safety features. Currently, there are medical devices with the latest and new technology in a smaller size and require only a low voltage current supply. For example, a traditional, more giant ultrasound machine can replace a mobile scan during Focussed Assessment with Sonography in Trauma (FAST scan). A medical device is altered to reduce the risk of electrical injury or accident. This can be done by placing the device at a safer site. Modifying the location can prevent any fall of the device, damage to the wire, and plug or contact with water. For example, multiple cables in the floor can cause fall or contact of worker with the current. Therefore, it is essential to design, so the wires are safely corded together and arranged at the side. A prevention program for the healthcare workers is needed to hinder any electrical injury or accident. A comprehensive approach assesses the department's OSH policy and OSH committee role. Besides that, analysing notification of accidents and injuries at the workplace will give additional information to sketch the program. These analyses include pitfalls and suggestions to improve and prevent future incidents. A health surveillance program can be conducted to ensure healthcare workers are in good condition to full fill their job tasks as stated in the job description. Workers with abnormal results must be referred and assessed by Occupational Health Doctor (OHD) to get clearance fitness to work (FTW).

Meanwhile, workers who sustain injury must return to work (RTW). RTW will ensure the capability of the worker to complete the job task and not put himself or others in danger. Compensation also can be applied to aid injured workers. Training and awareness are essential aspects of the prevention program. All new workers at the department must undergo orientation at the department. This includes fire floor plan, fire extinguisher availability, reporting accident and incidents, auditing workstations, etc. The audit's finding needs to be presented during the OSH committee meeting for the solution and improvement action. PPE's requirement depends on the workstation and hazard exposure. PPEs wore concurrently to protect against other hazards, such as biological and chemical. Nevertheless, it is advisable to keep the PPE's dry as water is a good conductor for electrical current. Health surveillance is an examination and investigation to detect exposure levels, early biological monitoring, and biological effect monitoring. Medical surveillance enquires about symptoms of occupational disease and reviews of records and occupational history.

Based on the findings from workplace assignments, regular health surveillance programs are highly recommended in the workplace. The basis of the health surveillance program is primary, secondary, and tertiary intervention. The direct intervention is for specific protection and health promotion. It is done to identify the hazard, assess the risk, and control it. It must be done regularly to make early corrective actions possible to eliminate the source. The specific protection can be done by pre-employment medical screening. Regarding the selected work unit, the fieldwork unit, essential blood investigation (FBC, FBS, Hyperlipidaemia) estimation is necessary to get a baseline because of risk to non-communicable disease that potential can influence work performance. Besides that, good history includes past medical history, past surgical history, occupational history, and physical examination, particularly looking at the central and peripheral nervous system and specific investigations. The person in charge plays a vital role in educating and promoting health to the supporting staff regularly, especially to those directly involved in possible hazards in the workplace. Secondary prevention is mainly for early detection of disease and early treatment. It is implemented when an incident has already happened. The person in charge can arrange for medical surveillance to the staff. Medical surveillance aims to monitor the team's health exposed to the health hazards at regular intervals. The components include biological monitoring, biological effect monitoring, and health effect monitoring on their hazard. In medical surveillance, the cause and effect must be investigated once an incident happens. Tertiary prevention is implemented when the disease pathology is already there for those exposed to occupational hazards. Its main aim is to limit disability and offer rehabilitation depending on disease or disability. After the medical removal protection (MRP) period and rest from the workplace, OHD shall reassess these employees for the Return to Work (RTW) program when the workplace hygiene is safe and healthy.

To the best of our knowledge, limited studies available to depict importance of managing electrical hazard at healthcare settings. Currently available articles focus more on general occupational safety and health cultures practices. Scanty of researchers conducted study on exploring knowledge, attitude, and practice managing electrical hazard and its health effect particularly in healthcare procedures. This is because of the complexity of the working condition and risk factors. In addition, fast moving technology innovation with latest electrical equipment applied in medical care and diagnosis were under reviewed for occupational safety and health measures. Healthcare workers sustained electrical injury even before detailed assessment conducted or user review collected. Hence, for future studies, we would like to urge occupational safety and health unit at healthcare setting to intensity surveillance activities for electrical injury among healthcare workers. These finding will compliment medical device industry to market safer equipment's for healthcare workers to operate. Subsequently, relevant preventive measure can be adopted and adapt into practice to hinder serious consequences for the healthcare workers as well as organization.

## 5 Conclusion

Electrical hazard is a significant threat in hospitals that require sustainable assessment and improvement. Hence, workplace assessment and medical surveillance systems are often overlooked, and employees fail to comply with the necessary measures that have been instructed at the workplace. This is attributed to a lack of knowledge regarding occupational safety and health and a lack of awareness regarding its importance. The consequences of neglecting these assessments may result in health deterioration, low work quality, and may also cause disability and impairment. Hospitals should adopt workplace assessments to improve the overall health of all the workers, leading to increased productivity in terms of quality in health service. Therefore, the recommendations outlined above are practical and feasible, and the implementation of these suggested recommendations will hopefully result in a safer and healthier working environment.

## Ethical approval

No ethical approval is required for this review.

## Sources of funding

This review study did not receive any specific grant from funding agencies in the public, commercial, or non-profit sectors.

## Author contribution

Loganathan Salvaraji-study concept or design, Mohammad Saffree Jeffree-study concept or design, Khamisah Awang Lukman-writing the paper, Sahipudin Saupin-study concept or design, Richard Avoi-writing the paper.

## Registration of research studies

Not applicable as it is a review and does not involve any new data collection from healthcare workers**.**

## Guarantor

Loganathan Salvaraji.

## Consent

Not applicable as it is a review and does not require patient details.

## Provenance and peer review

Not commissioned, externally peer reviewed.

## Declaration of competing interest

There is no conflict of interest in this study.
